# Compositional Sequence Generation in the Entorhinal–Hippocampal System

**DOI:** 10.3390/e24121791

**Published:** 2022-12-08

**Authors:** Daniel C. McNamee, Kimberly L. Stachenfeld, Matthew M. Botvinick, Samuel J. Gershman

**Affiliations:** 1 Neuroscience Programme, Champalimaud Research, 1400-038 Lisbon, Portugal; 2 Google DeepMind, London N1C 4DN, UK; 3 Gatsby Computational Neuroscience Unit, University College London, London W1T 4JG, UK; 4 Department of Psychology and Center for Brain Science, Harvard University, Cambridge, MA 02138, USA; 5 Center for Brains, Minds and Machines, MIT, Cambridge, MA 02139, USA

**Keywords:** compositionality, generative models, entorhinal cortex, hippocampus

## Abstract

Neurons in the medial entorhinal cortex exhibit multiple, periodically organized, firing fields which collectively appear to form an internal representation of space. Neuroimaging data suggest that this grid coding is also present in other cortical areas such as the prefrontal cortex, indicating that it may be a general principle of neural functionality in the brain. In a recent analysis through the lens of dynamical systems theory, we showed how grid coding can lead to the generation of a diversity of empirically observed sequential reactivations of hippocampal place cells corresponding to traversals of cognitive maps. Here, we extend this sequence generation model by describing how the synthesis of multiple dynamical systems can support compositional cognitive computations. To empirically validate the model, we simulate two experiments demonstrating compositionality in space or in time during sequence generation. Finally, we describe several neural network architectures supporting various types of compositionality based on grid coding and highlight connections to recent work in machine learning leveraging analogous techniques.

## 1. Introduction

The generation of new knowledge via the composition of multiple informative elements is a hallmark of natural intelligence and underpins a variety of sophisticated cognitive processes [1,2,3,4,5,6]. Compositionality enables complex representations to be formed combinatorially from simpler components efficiently and flexibly. This concept has been successfully applied by machine learning algorithms to problems ranging from control to vision and language [7,8,9,10]. We consider how compositionality may be achieved in neural circuitry, a long-standing problem in cognitive neuroscience [11,12,13]. In particular, we focus on compositionality in the context of internal simulations of dynamical systems and apply our model to spatial tasks in order to make contact with neural data [14].

Given its critical contribution to a variety of cognitive processes and capacity for sophisticated relational representations in the form of cognitive maps, we focus on the entorhinal–hippocampal circuit (EHC). The EHC is thought to contribute, in particular, to cognitive processes that rely on novel compositions of sequential information, such as imagination [15,16], transitive inference [17,18], novel one-shot associations [19], factorized replay [20] and spatial planning [21,22]. Although there is evidence that deep neural networks implicitly use compositional mechanisms to achieve their high performance in complex cognitive problems such as natural language processing [6,10], we have a limited understanding regarding how compositionality is implemented in specific neural systems such as the EHC, of which we have detailed knowledge [23]. Furthermore, data from several experiments hint at sophisticated regulatory and combinatorial roles for the medial entorhinal cortex (mEC) with respect to downstream activity in hippocampus (HC). In particular, mEC input is necessary for the temporal organization of hippocampal reactivations [24] and is causally involved in the concatenation of sharp-wave ripples (SWRs) in a form of sequential compositional replay known as extended replay [25].

In order to address this challenge, we develop a model of compositional sequence generation in the EHC, whereby distinct grid cell populations in the mEC are coordinated in order to configure sequential reactivations in the HC. The sequential reactivation of hippocampal representations, purportedly from a cognitive map, is thought to be an important algorithmic substrate in a variety of cognitive processes and has been conceptualized as the central role of hippocampal functionality [26]. In this work, we extend a recent theory of mEC grid cells as regulators of sequence generation which modulate the statistics and structure of replay including generating normative nonsequential activity patterns [27]. This model shows that grid cell populations can be distributively modulated across multiple spatial scales to parameterize different regimes of sequence generation within the EHC. In this work, we demonstrate that grid cells provide a representational substrate by which cognitive maps may be combinatorially expressed and naturally modulated for compositional sequence generation. Our analyses are directly inspired by algebraic formulas drawn from the theory of Lie groups and Lie algebras [28]. In simulation, we demonstrate that this mechanism is capable of recapitulating several empirical results regarding hippocampal reactivations, which we interpret computationally as different forms of compositional simulation.

**Figure 1:**
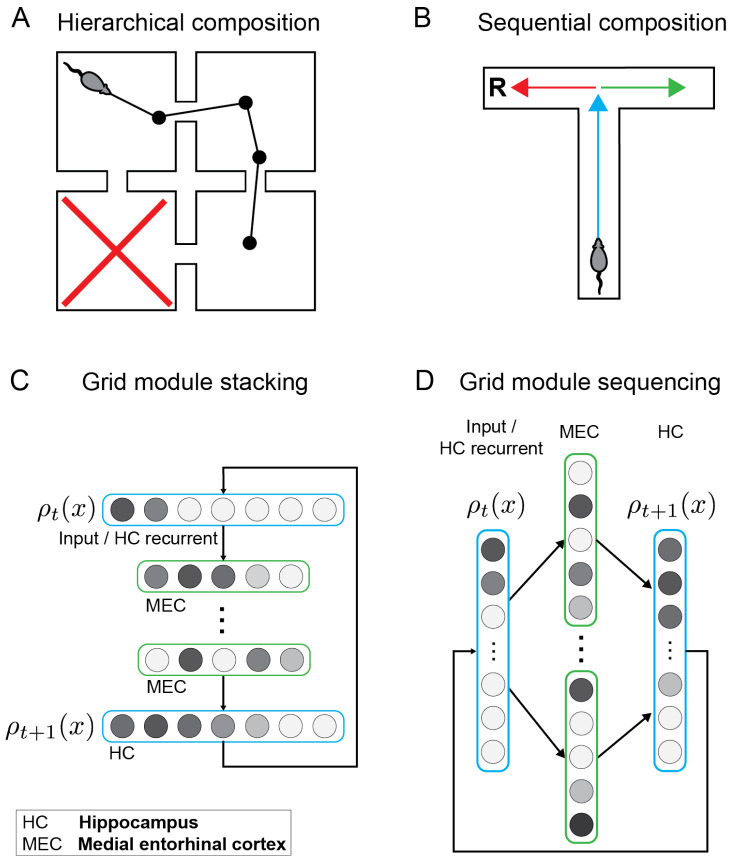
Distinct forms of composition in dynamical systems, hierarchical and sequential, and the associated proposed neural architectures in the entorhinal–hippocampal circuit. (**A**). Hierarchical composition. A rodent performing directed exploration searching for food in a four-room grid world. The rodent is presented with a cue that indicates that the food is not located in the bottom-left room (marked by a red X). How can this information be combined with an internal representation of the environment to generate efficient exploratory trajectories which avoid the bottom-left room? (**B**). Sequential composition. In this T-maze, optimal trajectories may be efficiently constructed by combining abstract behavioral components represented by the colored arrows. For example, a combination of the blue and red components are required in order to access the reward **R**. (**C**). Circuit diagram of grid module stacking for hierarchical composition. Each circle represents a network unit corresponding to a representative cell drawn from a distinct grid module. Grayscale coloring of neural units indicates variations in level of activation. Dynamical systems, encoded in separate grid modules (green), may be combined in a deep network architecture where each “hidden” layer encodes each of the distinct dynamical systems. (**D**). Circuit diagram of grid module sequencing for sequential composition. In contrast to grid stacking which manifests as spatially compositional sequence generation, grid sequencing corresponds to a temporal composition. At any time step, sequence generation under grid stacking is sensitive to all of the composed dynamical systems simultaneously, while only one dynamical system is active at any given time with grid sequencing whereby cells activate sequentially, consistent with entorhinal replay [31]. Note that grid stacking and grid sequencing are not mutually exclusive and potentially could be combined.

We demonstrate variations of our compositional generator framework within the context of three cognitive functions. First, in a simple adaptive exploration paradigm within a four-room grid world (Figure 1A), we show how to combine dynamics models corresponding to random exploration and directed exploration. Second, we show how concatenated sequences may be efficiently generated (Figure 1B) and suggest how this may underpin extended replay in ripple bursts [25]. Third, we demonstrate how sequence dynamics may be simultaneously controlled at different levels of hierarchical abstraction by distinct generators and show how this model explains so-called event-specific rate remapping of episodic experiences in the hippocampus [29].

Our technical contribution is a complete elaboration regarding how to generate samples from arbitrary combinations of dynamical systems using a network model of the EHC for which we provide a self-contained introduction [27]. Analytically, this is a delicate operation. A naive approach such as averaging transition models leads to unstable dynamics, and simple concatenation is not sufficient in general. In particular, a key challenge in this endeavor is to understand the commutation relationship between generators for distinct dynamical systems. Relatively simple dynamics in homogeneous state-spaces such as an open arena are commutative and therefore, the order of composition is irrelevant. However, many combinations of dynamical systems do not commute. That is, sampling a transition in one dynamical system and then the other is not equivalent to sampling each system in the reverse order. For example, in three-dimensional geometry, rotations are not commutative, which has fundamental implications for sensorimotor mechanisms including visual processing [30]. When getting dressed, it does not matter the order in which trousers and socks are put on however the order is important for a shirt and a jacket or socks and shoes. In the game of chess, a bishop move and a pawn move may not commute if the pawn blocks the bishop’s path. We draw on the theory of Lie algebras in order to address the challenge of composing noncommutative generators [28]. We finish by outlining possible directions for future work in theory and experiment, as well as highlighting connections to other models in the neuroscience and machine learning literature.

## 2. Methods

### 2.1. Cognitive Generators

In these sections, we provide a self-contained introduction to the cognitive generator theory for sequence generation in the entorhinal–hippocampal circuit [27]. We consider the problem of sampling sequences from continuous-time Markov processes ${\left\{X_{t}\right\}}_{t\in R}$ [32]. Such processes characterize how a state variable x∈X evolves over time under noisy dynamics. We denote the state at a particular time *t* as $x_{t}$ and conceptualize this as an internal state represented by a cognitive process unfolding in time. The state variable may, for example, reflect a position in an environment during a planning process or a particular memory being retrieved. The marginal probability of the random process generating a particular state $x_{i}$ at time *t* is denoted $\rho_{t}\left(x_{i}\right):=P\;\left(X_{t}=x_{i}\right)$ and $\rho_{t}$ constitutes a vector of state probabilities. Such a stochastic process is compactly specified by a master equation [33]:

(1) $$\tau \dot{\rho }=\rho O,$$

where the notation $\dot{\rho }$ indicates the time derivative of ρ and τ is a time constant. This equation describes the time evolution of the state probability vector ρ. The matrix *O*, known as the *infinitesimal generator*, defines the state dynamics at very short timescales:

(2) $$O_{ij}:=\lim_{\Delta t\to 0}\frac{P\;\left(X_{t+\Delta t}=x_{j}|X_{t}=x_{i}\right)}{\Delta t}.$$

The differential Equation (1) can be solved analytically to describe the density $\rho_{\Delta t}$ at an arbitrary time in the future, given an initial state distribution $\rho_{0}$ [32,33]:

(3) $$\rho_{\Delta t}=\rho_{0}e^{\tau^{-1}\Delta tO}.$$

This equation shows that the state probability row vector $\rho_{\Delta t}$ at time Δt is the product of the prior state probability row vector $\rho_{0}$ at time 0 and the matrix exponential of the infinitesimal generator *O*. Intuitively, this equation “starts from” the prior state density $\rho_{0}$ and uses the generator *O* to iterate the state probabilities forward in time until timepoint Δt, at a speed that is regulated by τ. By definition of *O*, $e^{\tau^{-1}\Delta tO}$ is a state transition matrix for all time intervals Δt≥0.

### 2.2. Sequence Sampling

Fixing Δt=1 for a single time step, the propagator $P_{\tau }=e^{\tau^{-1}O}$ can be applied iteratively to generate state distributions on successive time steps via

(4) $$\rho_{t+1}=\rho_{t}P_{\tau }.$$

State sequences characterizing the simulated evolution of the system can therefore be generated by recursively applying this propagator $P_{\tau }$ and sampling

(5) $$x_{t}∼e_{x_{t-1}}P_{\tau },$$

where $e_{x}$ is a one-hot row vector indicating that state *x* is active with probability one. This results in state sequences x that accurately reflect the generative distribution of sequences p(x) defined by the generator *O* and initialization $\rho_{0}$. By modulating the *tempo* τ, the speed of the generated sequence may be controlled. Increasing (or decreasing) τ results in a slower (or faster) time evolution.

### 2.3. Roles of Grid Cells and Place Cells in a Linear Feedback Network

The exponential $e^{M}$ of a matrix *M* is defined as [34]

(6) $$e^{M}=\sum_{n=0}^{\infty }\frac{M^{n}}{n!}.$$

Thus, directly computing the propagator

(7) $$P_{\tau }=\sum_{n=0}^{\infty }\frac{{\left(\tau^{-1}O\right)}^{n}}{n!}$$

is challenging since it requires an infinite sum of matrix powers. However, $P_{\tau }$ can be computed efficiently using a generator eigendecomposition O=GΛW (where *W* is the inverse matrix for *G*) as

(8) $$P_{\tau }=Ge^{\tau^{-1}\Lambda }W.$$

Since Λ is the diagonal matrix of *O*-eigenvalues, its exponentiation is trivially accomplished by exponentiating the eigenvalues separately along the diagonal ${\left[e^{\tau^{-1}\Lambda }\right]}_{kk}=e^{\tau^{-1}\lambda_{k}}$. Multiplication by *G* projects a state distribution $\rho_{t}$ on to the generator eigenvectors $\phi_{k}={\left[G\right]}_{\cdot k}$, which we refer to as the *spectral components* of the propagator. We use the term “spectral” to refer to a basis which diagonalizes the generator. Although we use simple eigendecompositions to demonstrate our compositional model here, spectral components may be computed based on the imposition of additional constraints, such as non-negativity, for further biological realism [27]. In this spectral representation, time rescaling simply corresponds to parametrically varying the *tempo* parameter according to the *power spectrum*

(9) $$s_{\tau }\left(\lambda \right)=e^{\tau^{-1}\lambda },$$

where λ corresponds to an eigenvalue associated with a particular eigenvector of *O*.

In previous work [27], it was also pointed out how this power spectrum may be parametrically modulated to produce qualitatively different forms of sequence generation. In particular, superdiffusive sequences, which are distinguished by occasional jumps between activated positions, may be generated by varying a stability parameter α to values less than 1 according to

(10) $$s_{\tau ,\alpha }\left(\lambda \right)=e^{-\tau^{-1}{\left|\lambda \right|}^{\alpha }}.$$

Furthermore, motivated by the normative objective of maximizing hippocampal sampling efficiency, nonparametric modifications to the power spectrum led to the production of nonsequential patterns of replay whereby successive hippocampal reactivations did not encode adjacent locations in the associated cognitive map [27]. In this manuscript, simulations relied on parametric variations in the power spectrum only with the tempo τ and stability α parameters fixed to the default values of τ=1 and α=1 (diffusive sampling) or α=0.5 (superdiffusive sampling). All model predictions compared to data were robust with respect to variations in this parametrization.

We now describe how these computations may be embedded within a fully connected linear network model with recurrent feedback [27]. Note that this simplified neural model is designed to establish a direct correspondence to the equations previously elaborated (Equations (5), (8) and (9)); however, further refinements may be included in order to reflect these computations within a continuous attractor network model [27]. The input state density vector $\rho_{0}$ is encoded in a population of hippocampal place cells (i.e., the firing rate of each place cell encodes the probability of occupying its preferred spatial location during sequence generation) or is presumed to be communicated from higher-order cortices. This representation inputs to a grid cell population with synaptic weights defined by the matrix *G*. Each column of *G* corresponds to a separate grid cell which is representative of a distinct grid module. Effectively, the output of this computation is a representation of the input spatial distribution in a spectral basis of the associated generator. The second synaptic weight matrix *W* recurrently maps this spectral representation back into the input space. By modulating the gain on the output of the second layer according to the power spectrum *s*, the network can control how far into the future those state transitions are generated. Within our neural model, we hypothesize that this may be accomplished by neuromodulatory gain control or grid rescaling [35]. The generator model proposes that grid cells serve as a basis set for infinitesimal generators of dynamical systems [27]. The compositional architectures elaborated in the present manuscript are variations on this network model (Figure 1C,D). For example, in the stacking architecture (Figure 1C), we show how deeper networks with multiple layers of grid cells can generate compositional sequences.

### 2.4. Propagator Composition

Within our framework, the simplest compositional mechanism is to concatenate *n* propagators $P_{1},\ldots ,P_{n}$ via

(11) $$\begin{array}{ccc} \rho_{t} & = & \rho_{0}P_{1}\cdots P_{n} \end{array}$$

(12) $$\begin{array}{ccc} \rho_{t} & = & \rho_{0}\prod_{i=1}^{n}G_{i}e^{t\Lambda_{i}}W_{i}, \end{array}$$

where we have used the corresponding generator decompositions $O_{i}=G_{i}\Lambda_{i}W_{i}$. Logically, this composition motif corresponds to an AND operation across propagators, which we denote $P_{1}\wedge \cdots \wedge P_{n}$. That is, sequence generation using the propagator composition (Equation (12)) results in sequences reflecting the dynamics associated with all propagators. If the propagators do not commute (i.e., if $[P_{i},P_{j}]\neq 0$ for any i,j) then the order of the propagators matters. We describe how noncommutative propagators may be composed in Appendix A.1. Alternative approaches to composing dynamical representations are available at the level of generators, which we elaborate in the next section.

### 2.5. Generator Composition

Any non-negative linear combination of two generators, say $O=\beta_{1}O_{1}+\beta_{2}O_{2}$, is also a generator [32]. This compositional generator defines a new dynamical system according to

(13) $$\begin{array}{ccc} \dot{\rho } & = & \rho O\\ & = & \rho \left[O_{1}+O_{2}\right] \end{array}$$

(14) $$\begin{array}{ccc} \rho_{t} & = & \rho_{0}e^{t\left[O_{1}+O_{2}\right]} \end{array}$$

More generally, compositional processing is described by the compositional master equation:

(15) $$\dot{\rho }=\rho \left(\sum_{i=1}^{n}w_{i}O_{i}\right)$$

which admits the compositional propagator as a solution:

(16) $$\rho_{t}=\rho_{0}e^{t\left(\sum_{i=1}^{n}w_{i}O_{i}\right)}.$$

The state-space dynamics described by the compositional propagator (Equation (16)) reflects the weighted contribution of each of the propagators $O_{i}$. The matrix exponential calculation required by the solution (Equation (16)) may be challenging to compute in general. This is due to the fact that, if some of the generators do not commute, then they cannot be simultaneously diagonalized; thus, the matrix exponential cannot be computed efficiently in a similar fashion to the case of a single generator (Equation (8)). An inflexible solution is to construct a specialized generator combining the contributions of the generators to be composed. We refer to this as *conjunctive composition* (Appendix A.3.1). In contrast, we demonstrate a flexible approach whereby the higher-order commutation relations between noncommutative generators are used to form a distinct cognitive *interface generator*, which encodes the appropriate higher-order interactions between noncommutative generators.

In summary, we lay out three computational techniques for flexibly composing two or more generators hierarchically, which we refer to as the *commutative composition* (Appendix A.2), *conjunctive composition* (Appendix A.3.1) and *interfacing composition* techniques (Appendix A.3.2). While the former is appropriate for composing commutative generators, the latter flexibly composes noncommutative generators. The latter include rotations in three dimensions, or rotations and translations, which are necessary when internally modeling sensorimotor interactions with our physical environments, e.g., during reaching or visually guided movements [30]. These commutation techniques are neurally realized in the grid stacking architecture (Figure 1C). In the description for a simulated example for our model (Section 3.2), we describe how an alternative approach may be leveraged to produce sequential composition in a generator sequencing architecture (Figure 1D) inspired by entorhinal replay [31].

## 3. Results

### 3.1. Composing Environment Information for Directed Exploratory Trajectories

Humans are capable of integrating sensory cues and structural knowledge of an environment to generate sophisticated directed exploration strategies [36,37]. Indeed, situated tasks encountered in real-world environments are often specified using several sources of information and burdened with multiple constraints. Consider finding a route to your favorite restaurant in a city. Novel information regarding roadworks blocking a major intersection can be rapidly fused with an established representation of the city structure in order to support adaptive spatial navigation. With respect to the hippocampus, this motivates the investigation of how multiple cognitive maps (each representing a different layer of information about the same environment) can be composed into a single representation useful for downstream computation. We describe how this can be accomplished mechanistically using generator compositionality. This mechanism accounts for the flexible adaptation of policies to changes in the environment structure, goals and other sources of information. Such a mechanism may be used, for example, to shift a random exploratory process to a directed search strategy [37] or to encode a taxic gradient [38].

**Figure 2:**
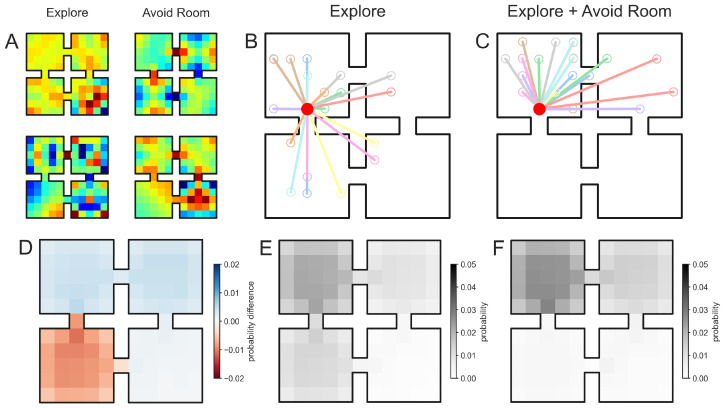
Neurocompositional mechanism for automatically integrating knowledge into exploratory sequence generation. (**A**). Example generator components for the “Explore” (left) and “Avoid Room” generators (right). (**B**). In a four-room environment, exploratory steps (20 samples represented by colored lines) are generated from an initial position (red) in the top-left room. (**C**). We composed (Equation (12)) an “Explore” propagator with an “Avoid Room” propagator which instructed the sequence generation process not to sample the bottom-left room as seen in 20 sampled exploratory steps. (**D**). The difference in spatial propagation densities generated by the “Explore” generator and “Explore + Avoid Room” compositional generator. In particular, red indicates that the probability of sampling this position is reduced in the compositional architecture due to the “Avoid Room” generator. (**E**,**F**). The spatial propagation densities for the “Explore” and “Explore + Avoid Room” sequence generators, respectively. As expected theoretically, sequence generation avoids sampling the bottom-left room.

We use our model to simulate an example whereby an agent has learned that a goal is not in a particular room of a four-room environment (lower-left room in Figure 2C), thus the agent should not generate sequences which sample from that room. Stacking the propagator of a random exploration generator $O_{\mathrm{explore}}$ (corresponding to a random walk process) with that of an “avoid room” generator $O_{\mathrm{avoid}}$ in a two-layer entorhinal–hippocampal network (Figure 1C) generates the requisite trajectories (Figure 2C) in contrast to the same network but with the “avoid room” propagator removed (Figure 2B). The “avoid room” generator $O_{\mathrm{avoid}}$ was constructed by modifying a random walk generator such that rows of the generator corresponding to states *s* in the avoided room were scaled according to $O_{s\cdot }\leftarrow cO_{s\cdot }$, where *v* is a free parameter such that if c=1 the room is sampled during sequence generation and as *c* increases the room becomes increasingly avoided. From a stochastic processes perspective, this generator modification corresponds to reducing the dwell time specifically for states in this room to the point that the time discretized sampling through the EHC tends not to activate these states [32]. A similar mechanism (though scaling inversely) was previously proposed to model the attraction of hippocampal trajectory events to goal locations [27]. The spectral components encoding the “Explore” $O_{\mathrm{explore}}$ and “Avoid Room” $O_{\mathrm{avoid}}$ generators exhibit heterogeneous multifield activation patterns with variations in peak firing rates [39].

### 3.2. Combining Generators for Sequential Compositional Replay

**Figure 3:**
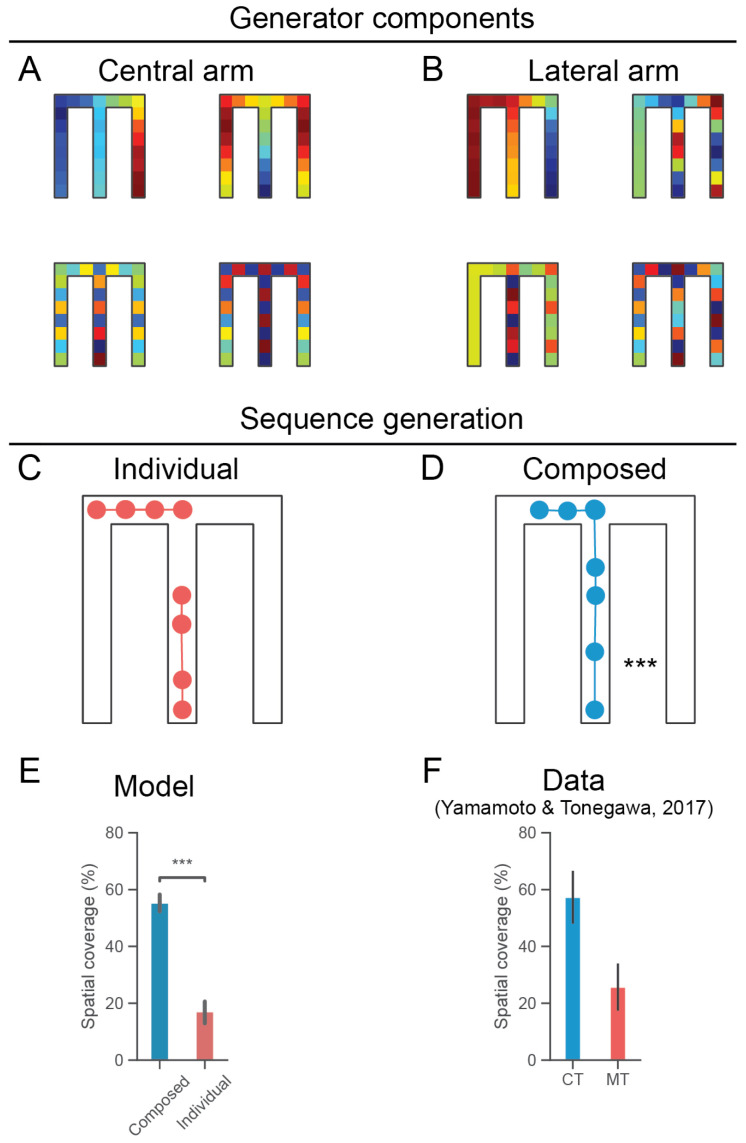
(**A**). Spectral components of the generator matrix which generates directed sequences through the central corridor. With respect to the activity profiles of grid cells, red (blue) reflects higher (lower) activity respectively. (**B**). Spectral components of the generator matrix which generates directed sequences through the lateral arm (specifically, the left arm). Note that these generator components indicate variable grid cell activity profiles throughout the state-space beyond the locales with directed dynamics (i.e., central arm or lateral arm). (**C**). Two separate sequences are generated following initialization in the central corridor and at the junction (red). (**D**). Grid modules are combined sequentially (Figure 1D) in order to form a compositional propagator generating extended sequences (blue). (**E**). Following the analysis of [25], we compared the spatial coverages of the individual (red) and composed (blue) sequences. This is the spatial extent covered by the generated sequences as a percentage of the shortest path from the start location in the central corridor to the end of either arm (where rewards were located in the corresponding experiments). The spatial coverages of the composed sequences were significantly greater ($p<10^{-3}$, Mann-Whitney U test). Error bars indicate standard error of the mean. (**F**). Composed sequences covered a significantly greater extent of the environment similar to sharp-wave ripple bursts exhibiting extended replay, which require medial entorhinal input [25]. MT refers to mice in which neurotransmitter release from MECIII pyramidal cells to CA1 is inhibited; CT refers to control mice. Error bars indicate standard error of the mean.

Across several experiments taking place in relatively large and complex environments, it has been observed that hippocampal reactivations can encode spatial sequences which are segmented according to an environment topology [40] and are sometimes concatenated to form extended trajectories across an environment [25]. This process of activation and coordination of sequences requires a sophisticated generative neural architecture. We reason that, given the causal influence of the mEC in the temporal organization of hippocampal dynamics, grid populations may contribute to this functionality [24]. In particular, that the grid sequencing network motif (Figure 1D) can support the temporal concatenation of sequence generation in hippocampus. Mathematically, consider the composition of generators corresponding to the central arm $O_{\mathrm{central}}$ and lateral arm $O_{\mathrm{lateral}}$ of a T-maze (Figure 1B):

(17) $$\begin{array}{ccc} \rho_{t+1} & = & \rho_{t}e^{\tau_{\mathrm{central}}O_{\mathrm{central}}+\tau_{\mathrm{lateral}}O_{\mathrm{lateral}}} \end{array}$$

where each generator encodes directed transitions in corresponding parts of the state-space and otherwise implements a random walk. For example, the $O_{\mathrm{central}}$ generator is directed in the central arm only. We simulated this model in a classic task environment in which a rodent begins in the central arm and then must make a binary choice whether to go left or right at the junction in order to acquire reward (Figure 3A). Sequences may be primarily driven by separate grid populations which encode directed dynamics for distinct topological segments of the maze (blue sequences in panel Figure 3A for the central arm and the left arm of the maze). Notably, grid cells coordinated with place cells during rest tended to be directionally modulated [41].The network architecture in Figure 1D facilitates the temporal composition of these sequences. That is, this network generates an extended sequence of place activations by first generating the sequence in the central arm, then generating the sequence in the left arm. The spatial coverage of the composed sequences was significantly higher than the individual segmented sequences as expected (Figure 3C). This is consistent with the analysis of extended replays as observed in ripple bursts [25]. Sharp-wave ripples occurring in bursts of up to three distinct events were recorded in CA1 during the quite awake state. The decoded trajectories were spatially contiguous, consistent with the idea that they were coordinated to represent longer trajectories across the environment (CT, Figure 3D). Notably, blocking the mEC input into hippocampal subfield CA1 using optogenetics disrupted the spatial coordination of replay across ripple bursts. This resulted in a spatially discontiguous, fragmented replay with a significantly smaller spatial coverage (MT, Figure 3D), consistent with our simulations.

### 3.3. Hierarchical Sequence Generation Results in Rate-Mapping Place Codes

It has been observed that neural population activity representing a putative cognitive map may also encode latent variables independent of their spatial sensitivities [42,43]. In particular, neural codes for spatiotemporal abstractions of experiences in a structured environment have been shown to emerge in an unsupervised manner in both human neuroimaging [44] and rodent electrophysiology [29]. Such a conjunctive coding of external spatial variables and internal abstracted variables facilitates the construction of sophisticated internal models which can support behavioral flexibility [45]. Indeed, many computational algorithms for behavioral control emphasize the use of spatiotemporal abstractions (e.g., hierarchical reinforcement learning) [46]. Naturally, these temporal abstractions may evolve in time at different timescales under distinct dynamical rules, thus motivating a compositional approach to sequence generation. For example, an animal may seek to maintain an internal representation of its present context over a longer timescale compared to encoding the detailed sensory representation of its current position during a traversal of an environment [47,48]. Thus, we suggest that in internally simulating trajectories traversing a cognitive map, abstract representations of context and the sensory-specific representations of position should be separately generated by distinct generators using different time constants in the sequence generation model [27]. We demonstrate the feasibility of such a mechanism using the stacked compositional architecture (Figure 1C) and compare the predicted population code to place cell recordings from a rodent navigation experiment designed to elicit the encoding of a latent environment variable [29].

**Figure 4:**
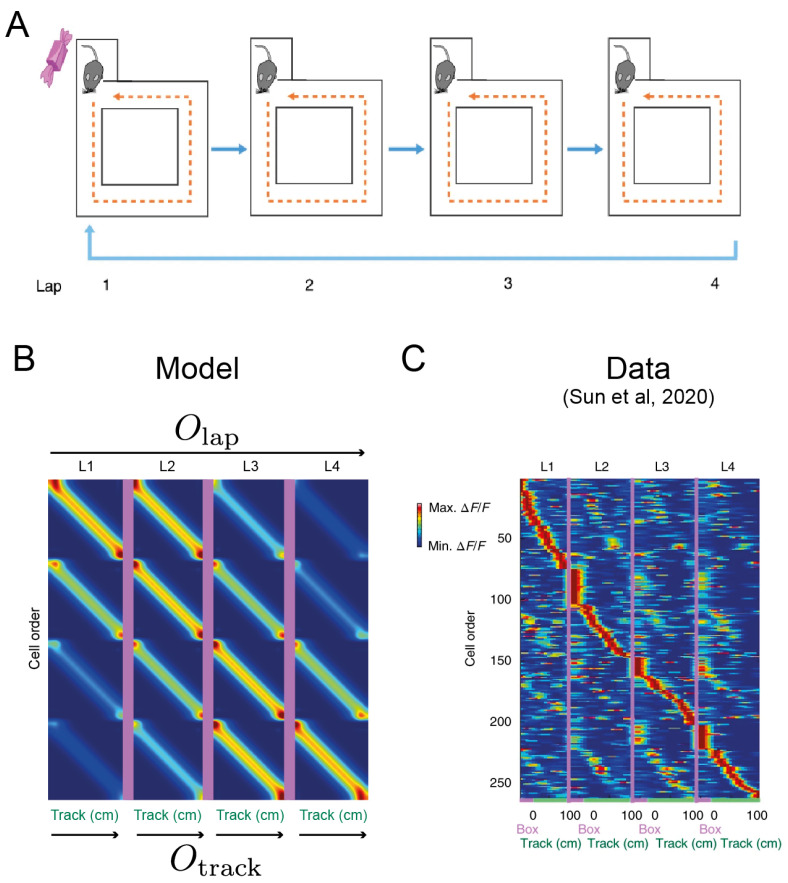
(**A**). The four-lap experiment in [29]. Mice traversed the track counterclockwise and only received a reward on the first traversal in each trial. (**B**). Model output from a stacked compositional architecture combining a lap generator $O_{\mathrm{lap}}$ and a track generator $O_{\mathrm{track}}$ (Figure 1C). (**C**). Event-specific rate remapping. A large proportion of place cells had a significantly higher firing rate on a particular lap of the track as measured by calcium imaging [29]. Thus, the place population rate coded for abstract events inferred from historical trajectories.

In this experiment, mice were required to traverse through the same square maze four times, though reward was only available (in a constant location) at the start of the first lap (Figure 4A). In addition to their spatial selectivity, the activity of a subpopulation of place cells was modulated by a preferred lap number. That is, the firing rates of these cells were higher on a particular lap in its associated place field (Figure 4B). This neural coding mechanism for spatiotemporal abstraction for distinct laps (or events more generally) is termed *event-specific rate remapping* (ESR) [29]. In order to account for the receptive field structure of ESR cells in our model, we simulated sequence generation using a stacked network composed of a box generator $O_{\mathrm{box}}$ and a lap generator $O_{\mathrm{lap}}$, which modulated the activation in a layer of ESR units which tiled a lap × box space (i.e., there was a distinct ESR unit for each combination of a place in the maze and a lap number). While the place code reflected an external environment variable, the lap number constituted an abstract latent code. The box generator $O_{\mathrm{box}}$ was biased to generate a counterclockwise traversal of the maze, while the $O_{\mathrm{lap}}$ generator controlled the iteration through the laps. Thus, the composition of these two generators led to the generation of multilap trajectories around the maze according to the dynamics:

(18) $$\begin{array}{ccc} \rho_{t+1} & = & \rho_{t}e^{\tau_{\mathrm{box}}O_{\mathrm{box}}+\tau_{\mathrm{lap}}O_{\mathrm{lap}}}. \end{array}$$

No higher-order corrections were required since these generators commuted $[O_{\mathrm{lap}},O_{\mathrm{box}}]=0$. We modeled the distribution of firing rates of each cell in the population using the propagated distribution initialized at each state in the lap × box space (Figure 4C). These predicted firing maps qualitatively matched those observed in the ESR cells (Figure 4B). In addition to their spatial selectivity, each unit had a preferred lap on which the firing rate was maximized. Each ESR cell in the HC layer had a preferred conjunction of lap and box position. Effectively, the distributed encoding of the composed generator in the mEC embedded the HC cells in a lap × box space such that the circuit dynamics generated the appropriate sequential activations corresponding to environment traversals (i.e., moving through the “same” track for four laps). However, this embedding also engendered a nonspatial generalization across lap space which resulted in a smaller number of activations of ESR cells on nonpreferred laps. Note that this effect emerges from the generic compositional architecture (Figure 1C) which can be applied to any combination of generators.

## 4. Discussion

We described and simulated a compositional generator mechanism for the EHC, which envisioned grid modules flexibly recruited and deployed based on ongoing cognitive requirements [27,49]. By comparing the model’s output to two datasets, it was shown how distinct network architectures related to compositional sequence generation in the temporal domain (in the form of extended replay) and hierarchically in the abstract spatial domain (resulting in event-specific rate remapping for a latent state). In the second application of our model, it was demonstrated that the composition of grid modules encoding a spatial generator and a lap generator may underpin the empirically observed phenomenon of event-specific rate remapping in hippocampal population activity. We further propose that this general computational motif need not be restricted to event-specific rate remapping per se; it may also be applied in alternate scenarios with different latent variables. However, our simulations diverge from the present experimental data in two ways. First, while ESR was observed in the hippocampus, no piece of data was acquired from the entorhinal cortex, which could be directly related to generator encoding [29]. This stands in contrast to our simulation of extended replay (Figure 3) which was shown to be causally dependent on the mEC input [25]. In particular, our model would predict distinct grid modules with activity profiles evolving over different timescales. However, this seems broadly consistent with the established role of the entorhinal cortex in regulating the temporal structure of hippocampal sequence generation [24]. Second, our model pertains to offline hippocampal reactivations whereas ESR was observed online as the rodent was traversing the environment [29]. It seems unlikely that ESR would be abolished in hippocampal replay given that a core feature of replay is the specific ordered reactivation of neural ensembles which were active in the awake mobile state. Thus, in order to create a bridge between theory and experiment, a natural avenue for further investigation would be to perform simultaneous recording in the entorhinal cortex and to establish whether ESR is preserved in hippocampal reactivations during sharp-wave ripples.

At the circuit level, the distinct network architectures associated with grid module composition may be translated into predictions for grid module organization in the mEC. For example, grid module stacking (Figure 1C) suggests that grid modules, encoding different generators or higher-order corrections, should be connected in a feedforward pathway. An empirical challenge to our model is the apparently tight coordination across grid modules. Even in the absence of sensory input, the correlational structure of population activity across grid modules is preserved [50]. We consider two possibilities. First, the tight correlational structure across grid modules has been observed in simple foraging tasks which obviate the need for cognitively sophisticated computations. Potentially, recording grid cells in relatively complex tasks may reveal flexible transients in grid module correlation patterns. Second, gridlike coding has been recorded in many cortical areas beyond the entorhinal cortex using functional magnetic resonance imaging [51]. It is possible that grid population activity in these regions do not exhibit a similarly tight correlational structure as in the entorhinal cortex and thus may more readily admit the type of compositional mechanisms we propose. Furthermore, the proposed neural architecture for noncommutative compositions based on Lie theory remains untested since neural recordings have not been made while noncommutative structural representations are experimentally manipulated. Potentially, this challenge may be overcome in rodent virtual reality paradigms whereby arbitrary rotations and translations in sensory input may be carefully controlled.

Technically, the sequence generation model is based on an exponential mapping from a representation of the infinitesimal transition structure of a dynamical system (encapsulated by a generator) to a distribution over states or positions at an arbitrary time point (i.e., the propagator) [32]. Using an efficient spectral encoding of these latent dynamics, multiple generators can be parsimoniously composed by stacking or sequencing in order to generate a variety of distinct distributions of state-space trajectories. The exponential mapping between generators and propagators is analogous to the exponential map in Lie theory connecting Lie groups to Lie algebras [28]. Generators form elements of a Lie algebra while propagators form the associated Lie group. Groups mathematically formalize the concept of symmetries in a space upon which they act via a group action. In the present context of dynamical systems, propagators correspond to a group of symmetries acting upon the set of distributions of states in the system. This perspective highlights connections to recent work in unsupervised learning seeking to extract disentangled representations from a given source of data [52,53], which coalesced around the concept of identifying independent symmetries within a dataset [54,55,56]. With respect to our work, each of these symmetries would be identified with a particular generator and associated grid module, which could then be generatively composed in the EHC architectures we have outlined. A possible line of future work is to extend such disentangled learning algorithms to the case of noncommutative generators (i.e., $[O_{1},O_{2}]\neq 0$) by adapting the Zassenhaus expansion (see Appendix A.3.2) for the deep learning context.

In contrast to unsupervised learning and the generative perspective presented here, alternative approaches to compositionality in cognitive maps have been developed based on reinforcement learning algorithms [4,9,57] centered around the linearization of the Bellman equation in Markov decision processes [4]. The most pertinent of these models constructed a variation on the successor representation [58], referred to as the default representation, which similarly exhibited periodically organized firing fields [57]. The default representation suffers a degree of inflexibility in its dependence on some aspects of an environment structure which may be circumvented by using the Woodbury matrix identity for compositionally constructing a cognitive map from component elements. However, a drawback of the Woodbury identity as a compositional mechanism is that the representation of each element depends on the other elements in the composition. For example, the vector representation $v_{A}$ of an element *A* depends on *B* in the composition $v_{A}\left(B\right)\circ v_{B}$ and must be modified if composing with *C* as in $v_{A}\left(C\right)\circ v_{C}$. This necessity for a multiplicity of representations of the same object undermines the flexibility and efficiency associated with compositional representation [1,2]. Indeed it is hypothesized that nonlinear computations would be required for a fully compositional theory of grid coding [57]. We suggest the higher-order terms in our model, inspired by the Zassenhaus expansion for exponential maps and reflected in generator interfaces in our model (Appendix A.3.2), may provide the requisite nonlinearities.

We focused on addressing how composition may manifest in the generation of sequential hippocampal reactivations given some of the known neural response profiles and circuitry of the entorhinal–hippocampal circuit. An important issue for future work is how the brain chooses which internal sequence generators to compose in what combinations, given a particular target cognitive computation. Given a multiplicity of distinct generators, the variety of different mechanisms by which they may be combined and the flexibility to compose regardless of the commutative structure of the associated dynamical systems indicate that a large combinatorial space of possible internal simulations may be activated. We suggest that the recruitment and organization of grid modules for composition may be mediated via higher-order cortical input according to cognitive control mechanisms. Indeed, recent human planning experiments have shown how humans manipulate their internal task representations in order to simplify the associated planning computations [59], thus demonstrating the utility of such a brain circuit mechanism. Potentially, such computational principles may be generalized beyond spatial cognition tasks to address more general compositional problems in cognition [60].

## Data Availability

Not applicable.

## Appendix A. Compositional Mechanisms

### Appendix A.1. Composing Noncommutative Propagators via Symmetrization

Consider the case of two noncommutative propagators *P* and $P^{\prime }$ such that $[P,P^{\prime }]\neq 0$ then $P\wedge P^{\prime }$ and $P^{\prime }\wedge P$ correspond to two distinct dynamical systems. However, a mixture of the two, $\frac{1}{2}P\wedge P^{\prime }+\frac{1}{2}P\wedge P^{\prime }$ symmetrizes the contribution of the component propagators, resulting in a composed propagator such that the order in which the propagators are considered is irrelevant. We denote this symmetrized propagator composition as

(A1) $$\begin{array}{ccc} P\wedge_{\mathrm{sym}}P^{\prime } & := & \frac{1}{2}P\wedge P^{\prime }+\frac{1}{2}P\wedge P^{\prime }\\ & = & \frac{1}{2}Ge^{t\Lambda }WG^{\prime }e^{t\Lambda^{\prime }}W^{\prime }+\frac{1}{2}G^{\prime }e^{t\Lambda^{\prime }}W^{\prime }Ge^{t\Lambda }W. \end{array}$$

### Appendix A.2. Commutative Composition for Compatible Generators

We define a *compatible composition* as the composition between two structural representations encoded within the same spectral representation. If two generators $O_{1}$ and $O_{2}$ commute, $O_{1}O_{2}=O_{2}O_{1}$, then they can be simultaneously diagonalized. That is, there exists matrices *G* and *W*, such that

(A2) $$\begin{array}{ccc} O_{1} & = & G\Lambda_{1}W \end{array}$$

(A3) $$\begin{array}{ccc} O_{2} & = & G\Lambda_{2}W. \end{array}$$

In this case, the matrix exponential formula (Equation (16)) reduces to

(A4) $$\begin{array}{ccc} e^{t\left[O_{1}+O_{2}\right]} & = & e^{tO_{1}}e^{tO_{2}}\\ & = & Ge^{t\Lambda_{1}}WGe^{t\Lambda_{2}}W\\ & = & Ge^{t\Lambda_{1}}e^{t\Lambda_{2}}W\\ & = & Ge^{t\left(\Lambda_{1}+\Lambda_{2}\right)}W \end{array}$$

Note that this implies that sequence generation based on the composition of any number of compatible generators may be accomplished by spectral modulation, thus, delivering an extremely efficient compositional mechanism.

### Appendix A.3. Noncommutative Composition for Generators

#### Appendix A.3.1. Conjunctive Generator Composition

In the propagation via *conjunction* approach, the generators to be combined, *O* and $O^{\prime }$, are replaced by their summary conjunctive generator $O_{C}:=O+O^{\prime }$. In this case, the propagator circuit makes use of a specialized eigendecomposition $O_{C}=G_{C}\Lambda_{C}W_{C}$ in order to precisely generate state-space dynamics according to the composition of *O* and $O^{\prime }$:

(A5) $$\begin{array}{c} \rho_{t}=\rho_{0}e^{t\left(O+O^{\prime }\right)}=\rho_{0}G_{C}e^{t\Lambda_{C}}W_{C}. \end{array}$$

Although suitable for composing noncommutative generators, this amalgamation of generators is inflexible; thus, one would expect such a representational strategy would only be employed for commonly encountered generative processing.

#### Appendix A.3.2. Interfaces for Noncommutative Generator Compositions

Consider two generators *O* and $O^{\prime }$ which do not commute (i.e., their Lie bracket is nonzero):

(A6) $$\left[O,O^{\prime }\right]:=OO^{\prime }-O^{\prime }O\neq 0.$$

This implies that *O* and $O^{\prime }$ cannot be simultaneously diagonalized and thus composed for sequence generation via spectral modulation (Equation (A4)). However, it is possible to approximate the composition of these generators using the Zassenhaus expansion [61,62]:

(A7) $$\begin{array}{cc} e^{X+Y} & =e^{X}e^{Y}\prod_{n=2}^{\infty }e^{Z_{n}\left(X,Y\right)}\\ \; & =e^{X}e^{Y}e^{Z_{2}\left(X,Y\right)}e^{Z_{3}\left(X,Y\right)}\cdots e^{Z_{n}\left(X,Y\right)}\cdots \end{array}$$

with terms

(A8) $$\begin{array}{ccc} Z_{2}\left(X,Y\right) & = & \frac{1}{2}\left[X,Y\right] \end{array}$$

(A9) $$\begin{array}{ccc} Z_{3}\left(X,Y\right) & = & \frac{1}{3}\left[Y,\left[X,Y\right]\right]+\frac{1}{6}\left[X,\left[X,Y\right]\right] \end{array}$$

(A10) $$\begin{array}{ccc} \cdots & = & \cdots \end{array}$$

which can be computed in various ways (e.g., via comparison with the Baker–Hausdorff–Campbell formula). The matrix exponential $e^{t(O+O^{\prime })}$ can then be approximated as:

(A11) $$\begin{array}{ccc} e^{t(O+O^{\prime })} & \approx & e^{tO}e^{tO^{\prime }}e^{t^{2}Z_{2}\left(O,O^{\prime }\right)}e^{t^{3}Z_{3}\left(O,O^{\prime }\right)}\cdots . \end{array}$$

Based on the eigendecomposition of the generators $O,O^{\prime },Z_{2},Z_{3},\ldots$, the propagator can then be expressed by concatenating the propagators associated with each of the composed generators along with commutators contributing higher-order corrections:

(A12) $$\begin{array}{ccc} \rho_{t} & = & \rho_{0}Ge^{t\Lambda }WG^{\prime }e^{t\Lambda^{\prime }}W^{\prime }G_{2}e^{t^{2}\Lambda_{2}}W_{2}\cdots . \end{array}$$

We refer to this as an *interfacing generator composition*. This method makes use of a series of spectral decompositions of generator commutation relations (captured by the Lie brackets) in order to approximate the matrix exponential $e^{t(O+O^{\prime })}$ to arbitrary precision. Note that the higher-order corrections (Equation (A11)) vanish when the Lie bracket is zero $[O_{1},O_{2}]=0$ for compatible composition and the relation $e^{t(O+O^{\prime })}=e^{tO}e^{tO^{\prime }}$ is exact.
